# Identity Threats as a Reason for Resistance to Artificial Intelligence: Survey Study With Medical Students and Professionals

**DOI:** 10.2196/28750

**Published:** 2022-03-23

**Authors:** Ekaterina Jussupow, Kai Spohrer, Armin Heinzl

**Affiliations:** 1 University of Mannheim Mannheim Germany; 2 Frankfurt School of Finance & Management Frankfurt Germany

**Keywords:** artificial intelligence, professional identity, identity threat, survey, resistance

## Abstract

**Background:**

Information systems based on artificial intelligence (AI) have increasingly spurred controversies among medical professionals as they start to outperform medical experts in tasks that previously required complex human reasoning. Prior research in other contexts has shown that such a technological disruption can result in professional identity threats and provoke negative attitudes and resistance to using technology. However, little is known about how AI systems evoke professional identity threats in medical professionals and under which conditions they actually provoke negative attitudes and resistance.

**Objective:**

The aim of this study is to investigate how medical professionals’ resistance to AI can be understood because of professional identity threats and temporal perceptions of AI systems. It examines the following two dimensions of medical professional identity threat: threats to physicians’ expert status (professional recognition) and threats to physicians’ role as an autonomous care provider (professional capabilities). This paper assesses whether these professional identity threats predict resistance to AI systems and change in importance under the conditions of varying professional experience and varying perceived temporal relevance of AI systems.

**Methods:**

We conducted 2 web-based surveys with 164 medical students and 42 experienced physicians across different specialties. The participants were provided with a vignette of a general medical AI system. We measured the experienced identity threats, resistance attitudes, and perceived temporal distance of AI. In a subsample, we collected additional data on the perceived identity enhancement to gain a better understanding of how the participants perceived the upcoming technological change as beyond a mere threat. Qualitative data were coded in a content analysis. Quantitative data were analyzed in regression analyses.

**Results:**

Both threats to professional recognition and threats to professional capabilities contributed to perceived self-threat and resistance to AI. Self-threat was negatively associated with resistance. Threats to professional capabilities directly affected resistance to AI, whereas the effect of threats to professional recognition was fully mediated through self-threat. Medical students experienced stronger identity threats and resistance to AI than medical professionals. The temporal distance of AI changed the importance of professional identity threats. If AI systems were perceived as relevant only in the distant future, the effect of threats to professional capabilities was weaker, whereas the effect of threats to professional recognition was stronger. The effect of threats remained robust after including perceived identity enhancement. The results show that the distinct dimensions of medical professional identity are affected by the upcoming technological change through AI.

**Conclusions:**

Our findings demonstrate that AI systems can be perceived as a threat to medical professional identity. Both threats to professional recognition and threats to professional capabilities contribute to resistance attitudes toward AI and need to be considered in the implementation of AI systems in clinical practice.

## Introduction

### Objective

Advances in machine learning and image recognition have driven the implementation of systems based on artificial intelligence (AI) in clinical practice. These systems are able to perform tasks commonly associated with intelligent beings, such as reasoning or learning from experience [1]. AI systems based on machine learning gain insight directly from large sets of data [2]. Such AI systems can, therefore, make more complex decisions and complete more complex tasks than can rule-based systems. Moreover, machine learning has the potential to improve AI system capabilities with growing amounts of training data. In medical disciplines such as radiology, AI systems already diagnose specific diseases in a manner comparable with that of experienced physicians [3]. Current AI systems provide diagnosis and treatment recommendations, facilitate access to information, and perform time-consuming routine tasks of physicians, such as the segmentation of radiological images [4]. In the light of an accelerated technological development, AI systems in health care are expected to pave the way for truly personalized medicine by augmenting medical decisions and helping physicians cope with increasing amounts of relevant data and growing numbers of medical guidelines [5]. In fact, future AI systems are expected to autonomously execute medical actions (eg, communicating results and making diagnosis decisions) as efficiently and accurately as expert physicians [3]. Thus, medical professionals will be able to delegate more complex medical tasks to those systems than to any traditional rule-based clinical decision support system [6].

Despite their benefits and the great expectations toward their future use, AI systems do not cause only positive reactions in medical professionals. Although nearly all current applications of AI are still limited to narrow use cases, the future potential of this technology has resulted in a discourse driven by a duality of hyped expectations and great fears [7]. Many physicians seem to be concerned about the changes that AI systems impose on their profession [8]. Such negative attitudes toward new technology can manifest as *resistance* attitudes, resulting in hesitation toward adopting a technology [9] and even in active resentment against using a technology in clinical practice [10]. Although multiple studies have investigated attitudes toward AI [11-13], they did not consider how resistance attitudes toward AI are formed. Specifically, they did not examine whether negative attitudes toward AI stem from the perception that AI is threatening the medical professional identity. Therefore, we aim to address the following research questions:

1. How is the medical professional identity threatened by AI systems?
2. Under which conditions do medical professional identity threats contribute to resistance to AI?

### Theoretical Background

*Professional identity* refers to *how professionals define themselves in terms of their work roles and provides an answer to the question* “Who am I as a professional?” [14]. In general, professionals strive to maintain a coherent and positive picture of themselves [15], resulting in a tendency to interpret experiences in identity-coherent ways. This helps individuals to adapt to social changes, such as technological innovations, and to create benefits from the positive identification with a social group. Professional identity can be considered as a combination of social identity [16] (ie, membership in a group of professionals) and personal identity (ie, the individual identification and enactment of professional roles) [15]. Medical professionals are known for their strong commitment to their professional identity, which is already developed in the early phases of their socialization and later refined through practical experience [17]. The medical profession’s group boundaries are very rigid and shaped by strong core values and ideals [18]. Medical professionals can, therefore, be seen as a *prototypical profession* [17], and their professional identity is particularly resilient to change [19].

*Identity threats* are “experiences appraised as indicating potential harm to the value, meanings, or enactment of an identity” [20]. Such experiences are potentially decreasing an identity’s value, eliminating a part of the identity, or disturbing the connection between an identity and the meaning the individual associates with the threatened identity [20]. In the following, we distinguish between two parts of individuals’ identity that can be threatened: personal identity and professional identity.

First, *self-threat* describes a context-independent threat to *personal identity* by challenging fundamental self-motives of distinctiveness, continuity, generalized self-efficacy, and self-esteem [21]. Self-threat can bias information processing, result in avoidance of threatening information [22-24], and adverse emotional reactions [25]. Recently, self-threat has been identified as an antecedent of resistance to technology [26].

Second, medical professionals can be threatened along different dimensions of their *professional identity*. Drawing on a synthesis of prior work (Multimedia Appendix 1 [9,10,14,27-34]), we differentiate between 2 dimensions of medical professional identity threats. First*, threats to professional recognition* refer to challenges to the expertise and status position of medical professionals [10,27]. Second, *threats to professional capabilities* refer to the enactment of roles related to the medical work itself. The latter include threats to the care provider role [28], autonomy [14,29-31], and professional control [9,32]. Multiple studies show that professional identity threats can stipulate resistance to new technologies and organizational change [10,28,35]; however, it is unclear how these threats manifest in the context of AI systems.

Multiple conditions do influence how medical professionals experience identity threat from AI systems. In this paper, we focus on the following two conditions: professional experience and the perceived temporal distance of AI. First, perceived experience influences how likely medical professionals perceive that AI systems can replace parts of their work. In particular, more experienced physicians believe that they have unique skills that AI systems cannot substitute, whereas novices have yet to develop those skills. Second, medical professionals might have different perceptions of how fast AI systems are implemented in clinical practice. Perceiving AI systems as temporally close and relevant in the immediate future suggests that AI systems will be seen as more influential on concrete medical work practices, thus threatening one’s professional capabilities. Conversely, if AI systems are perceived to be relevant only in the distant future, the perceived threat might be less specific to medical work practices but more relevant for the long-term reputation of medical professionals, thus threatening their professional recognition. Hence, the perceived temporal distance of AI systems could affect how relevant each dimension of the professional identity becomes [36].

## Methods

### Survey Design

We collected data in 2 waves of a web-based survey. The first wave survey mainly addressed medical students, whereas the second survey focused on experienced physicians from different specialties. All participants were provided with a vignette of an AI system named *Sherlock* (Textbox 1) that was based on the description of IBM Watson and was pretested with researchers and medical students. We selected this vignette because it depicts a general AI system in which the participants were familiar with because of the marketing efforts of the vendor. It does not limit the abilities of the AI to a specific medical specialty. The vignette was purposefully focused on the benefits of the system and evoked expectations of high accuracy to establish the picture of a powerful AI system that goes beyond extant narrow use cases and, thus, has the potential to be threatening. Then, control questions were included to ensure that participants associated AI with the vignette and that it was perceived as realistic. The vignette was pretested with medical students and professionals. We then asked an open question about the participants’ perceptions of the changes to their professional role caused by AI systems to gain qualitative insights into the perceived upcoming change of their identity. Afterward, we asked participants to complete the provided survey of experience, identity threat, resistance attitudes, and perceived temporal distance of AI systems.

**Vignette Sherlock—a general artificial intelligence system.**

**What is an intelligent clinical decision support software?**

- Physicians often need to quickly analyze all the information provided to make diagnoses and decisions about treatments. These decisions have far-reaching consequences for patients and yet often have to be made under time pressure and with incomplete information. This is where the Sherlock decision support software comes in.
- Sherlock can be used in different specialties but will be presented here as with the following example of an oncological system.
- Every physician has an iPad or a laptop through which he or she can access his or her electronic medical records. The “Ask Sherlock” button is integrated into each medical record. When the physician asks Sherlock, he or she receives a 1-page treatment recommendation with up to 40 pages of explanations as backup.
- With the help of artificial intelligence, Sherlock can integrate and compare millions of patient data to identify similarities and connections. In addition, Sherlock has been trained by renowned experts. On the basis of the evidence base and the expert training, Sherlock then generates treatment options. Sherlock then presents those treatment options ranked by appropriateness (recommended, for consideration, or not recommended) alongside key information from the medical record and relevant supporting articles from current research. As a result, the practicing physician can easily follow Sherlock's recommendations and directly access relevant articles or clinical data.
- Sherlock is already in use in some clinics and tests have shown that Sherlock's recommendations are 90% in line with the decisions of renowned tumor boards.

### Measures

We used a 4-item measure of self-threat [21] on a 6-point Likert scale and a 3-item measure of resistance [9]. Furthermore, we asked participants whether they perceived the change from AI systems as temporally close or distant by assessing the agreement to the following statements: “Such systems will only become relevant in the distant future,” “Such systems are unlikely to be implemented technically,” and “Such systems are too abstract and intangible for me.” We extended existing measures for threats toward professional recognition and professional capabilities following the procedure of MacKenzie et al [37], as outlined in Multimedia Appendix 2 [9,28,29,37-46]. For medical students, we also assessed positive expectations toward AI, which mirrored the negatively framed items that we used for identity threat (*identity enhancement*). We also included an open question about their expectations of how the medical role would change with the introduction of AI systems. As a control variable, we asked for participants’ familiarity with clinical decision support systems. Except items related to self-threat, all items were measured on a 5-point Likert scale from totally disagree to totally agree. Table 1 lists the survey items. The items for *identity enhancement* can be found in Multimedia Appendix 3 [47-50].

**Table 1:** Final list of items used for the hypothesis testing.^a^

Construct			Item
**Threats to professional recognition (self-developed from literature review)**			
	**Threat to expertise**		
		E2: I fear that when using the system, physicians may lose their expert status.	
		E3: I fear that when using the system, certain physician specializations can be replaced.	
	**Perceived threat to status position**		
		S1: I fear that when using the system, physicians’ position in the hospital hierarchy may be undermined.	
		S2: I fear that when using the system, physicians may have a lower professional status.	
		S4: I fear that the status of physicians, who use the system, may deteriorate within the physician community.	
**Threats to professional capabilities**			
	**Perceived threat to autonomy (adapted from Walter and Lopez [29])**		
		A1: I fear that when using the system physicians’ job autonomy may be reduced.	
		A3: I fear that physicians’ diagnostic and therapeutic decisions will be more monitored by nonphysicians.	
	**Perceived threat to professional influence**		
		I1: I fear that when using the system physicians may have less control over patient medical decisions.	
		I2: I fear that when using the system physicians may have less control over ordering patient tests.	
		I3: I fear that when using the system physicians may have less control over the distribution of scarce resources.	
	**Perceived threat to being a care provider (self-developed from literature review)**		
		C1: I fear that when using the system physicians have less influence on patient care.	
		C3: I fear that when using the system physicians are less able to treat their patients well.	
**Self-threat from AI^b^ (adapted from Murtagh et al [21])**			
			ST1: Using Sherlock undermines my sense of self-worth.
			ST2: Using Sherlock makes me feel less competent.
			ST3: Using Sherlock would have to change who I am.
			ST4: Using Sherlock makes me feel less unique as a person.
**Resistance to AI (adapted from Bhattacherjee and Hikmet [9])**			
			RC1: I do not want Sherlock to change the way I order patient tests.
			RC2: I do not want Sherlock to change the way I make clinical decisions.
			RC3: I do not want Sherlock to change the way I interact with other people on my job.
			RC4: Overall, I do not want Sherlock to change the way I currently work.
**Temporal distance of AI (self-developed)**			
			A1: Such systems will only become relevant in the distant future.
			A2: Such systems are unlikely be implemented technically.
			A3: Such systems are too abstract and intangible for me.
**Familiarity**			
			F1: I have never heard of such systems to I have heard a lot of such systems
			F2: I have never used such systems to I have used such systems quite often
			F3: I have never dealt with such systems to I have dealt with such systems in great detail.
			F4: I am not at all familiar with such systems to I am very familiar with such systems.

^a^Items with the identifiers E1, S3, A2, and C2 were removed because of measurement properties (see Multimedia Appendix 2).

^b^AI: artificial intelligence.

### Scale Validation

We validated the scales of professional identity threats in the sample of novices and experienced physicians and the corresponding identity enhancement values, as outlined in Multimedia Appendix 2. A confirmatory factor analysis with all measurement scales resulted in a good model fit. All scales displayed good psychometric properties, including reliability, convergent validity, and discriminant validity (Multimedia Appendix 2). The correlation between threats to professional recognition and professional capabilities was 0.64**,** which was smaller than the lowest square root of the average variance extracted of 0.77, indicating acceptable multicollinearity. Similarly, multicollinearity between self-threat and threats to professional recognition was acceptable with a correlation of 0.66 being lower than the lowest square root of the average variance extracted of self-threat. We accounted for potential common method bias in the survey design and through testing for a common method factor. The results indicated that common method bias is unlikely to have a strong impact on our results (see Multimedia Appendix 2 for details). The items of identity enhancement were combined into 1 factor because of the result of the exploratory factor analysis. All analyses were performed using SPSS (version 26; IBM Corporation) and Stata (version 16; StataCorp).

### Sample and Participants

A total sample of 227 novice and experienced physicians participated between fall 2017 and spring 2019. Participants were recruited from medical social media groups and by personal reference. After excluding participants because of failed comprehension checks or poor data quality (ie, very fast completion time or answers to the open question that were completely unrelated to the question), a total data sample of 206 participants was used for data analysis. Of these 206 participants, 164 (79.6%) were medical students and 42 (20.4%) participants were medical professionals across different specialties (see Table 2 for details on sample). We included both medical students and trained physicians in our sample for two reasons: First, especially inexperienced physicians are susceptible to the influence of technology [51]. They may thus provide valuable insight into the effects of AI systems. Second, particularly medical students face strong, long-term career consequences if AI systems alter the meaning of specific medical disciplines such as radiology. They are thus likely to cognitively engage with potential identity threats to make reasonable career decisions, for example, with regard to the specialty they pursue. Conversely, experienced medical professionals may have a more pronounced professional identity and may experience threats from AI systems differently.

**Table 2:** Sample properties of novice and experienced physicians.^a^

	Novice physicians	Experienced physicians
Total sample size, N	182	45
Sample used for data analysis, n (%)	164 (90.1)	42 (93.3)
Age (years), mean (SD)	24.65 (3.23)	39.57 (13.14)
Gender (female), n (%)	131 (72)	18 (40)
Experience (average)	Eighth semester	12.6 years of job experience

^a^The specialties of experienced physicians are presented at a later stage.

### Statistical Analysis

Our main outcome variable measures participants’ resistance toward using the *Sherlock* application. Because of differences in sample size between novice physicians (n=164) and experienced physicians (n=42), we conducted a nonparametric Mann–Whitney *U* test to test the differences between the 2 samples regarding resistance attitude and self-threat. We conducted seemingly unrelated regression analyses with self-threat and resistance as the dependent variables because we expected their error terms to be correlated. The predictors were included in a stepwise approach to assess how much additional variance they explain. To test the effect of perceived temporal distance of AI, we incorporated 2 interaction terms between temporal distance and the 2 dimensions of professional identity threats into the regression analysis. We conducted a mediation analysis following Hayes [52] with 10,000 bootstrap samples to test the mediating role of self-threat on resistance attitudes.

### Ethics Approval

Ethics approval was not sought or obtained from the Ethics Committee at the University of Mannheim, as an approval was not required under the institution’s current ethics statute.

## Results

### Descriptives and Group Differences

We find that both experienced and novice physicians perceived identity threats from the upcoming change from AI systems (Table 3). Novice physicians showed relatively high resistance to AI and self-threat, whereas experienced physicians showed slightly lower resistance and self-threat from AI. The group differences were significant for resistance (*P*=.005) and self-threat (*P*<.001). Novices perceived equally strong threats to their professional recognition (mean 3.05, SD 1.23) and professional capabilities (mean 3.25, SD 0.97), whereas experienced physicians perceived a stronger threat to their professional capabilities (mean 2.72, SD 1.18) than to their professional recognition (mean 2.38, SD 1.03). Group differences were statistically significant; novices experienced more threats to professional recognition (*P*<.001) and professional capabilities (*P*=.80) than experienced physicians. Similarly, novices reported AI systems as slightly more temporally distant than did experienced physicians (*P*=.08). Moreover, in the sample of experienced physicians, the experienced threats and resistance attitudes differed based on the medical specialty (Table 4). The descriptive statistics show, for example, that physicians in the psychiatry specialty reported stronger threats to professional recognition than to professional capabilities, whereas surgeons reported stronger threats to professional capabilities than to professional recognition. However, as the sample size was relatively small, more research is needed to fully understand the effects of different specialties on experienced identity threats.

**Table 3:** Mann–Whitney *U* test between novice and experienced physicians.^a^

	Novice physicians (n=164), mean (SD)	Experienced physicians (n=42), mean (SD)	Group differences	
			*Z* value	*P* value
Resistance to AI^b^	3.58 (0.97)	3.13 (0.97)	2.83	.005
Self-threat from AI	2.78 (1.36)	1.92 (0.91)	3.85	<.001
Threats to professional recognition	3.05 (1.23)	2.38 (1.03)	3.36	.001
Threats to professional capabilities	3.25 (0.97)	2.72 (1.18)	2.62	.01
Perceived temporal distance of AI	2.23 (0.84)	2.03 (0.94)	1.75	.08
Familiarity with AI	1.75 (0.89)	2.32 (0.77)	–4.28	<.001

^a^All items except self-threat were measured on a 5-point Likert scale and self-threat was measured on a 6-point scale ranging from strongly disagree to strongly agree.

^b^AI: artificial intelligence.

**Table 4:** Means (SDs) by specialty of experienced physicians (n=42).

Specialty	Values, mean (SD)				
	Self-threat from AI^a^	Resistance to AI	Temporal distance of AI	Threats to professional recognition from AI	Threats to professional capabilities from AI
Not specified (n=7)	2.32 (0.89)	3.71 (1.25)	1.71 (0.65)	2.48 (0.87)	3.18 (1.33)
Internal medicine (n=10)	2.05 (0.98)	3.15 (0.88)	1.83 (0.65)	2.28 (1.33)	2.62 (1.27)
General medicine (n=3)	1.92 (1.01)	2.92 (0.88)	2.78 (1.68)	3.06 (0.59)	3.57 (0.95)
Psychiatry (n=5)	1.55 (0.82)	2.70 (0.97)	1.53 (0.61)	2.50 (0.90)	1.94 (0.73)
Pediatrics (n=5)	2.05 (1.25)	2.80 (1.14)	2.13 (1.17)	2.97 (0.84)	3.00 (1.06)
Surgery (n=5)	1.45 (0.45)	3.10 (1.10)	2.07 (0.64)	1.53 (0.63)	2.11 (1.44)
Anesthesiology (n=3)	1.75 (0.90)	3.58 (0.63)	1.78 (0.38)	2.94 (1.08)	2.96 (0.83)
Others such as neurology and pathology (n=4)	1.94 (1.13)	2.81 (0.59)	3.08 (1.52)	2.40 (0.83)	2.96 (1.04)

^a^AI: artificial intelligence.

### Regression Analyses

Testing the relationships between different types of identity threat and resistance attitudes in the total sample (Tables 5 and 6; Multimedia Appendix 3), we found that perceived professional identity threats directly affected resistance attitudes and personal identity threat (self-threat). Both threats to professional recognition (*P*<.001) and threats to professional capabilities (*P*<.001) were significant predictors of self-threat (model 4a, Table 6). Moreover, we found that both professional identity threats contributed independently to resistance to change. However, threats to professional recognition predicted resistance only in isolation (Multimedia Appendix 2; *P*<.001) but not in combination with threats to professional capabilities (Multimedia Appendix 3, model 3b; *P*=.50). Hence, threats to professional capabilities overruled the impact of threats to professional recognition on resistance attitudes and significantly increased resistance (Multimedia Appendix 3, model 3b; *P*<.001). The findings suggest that threats to professional recognition are more strongly related to personal identity, whereas threats to professional capabilities are more strongly and directly related to resistance to change.

**Table 5:** Descriptive statistics of the measurement model (N=206).^a^

Variables	Mean (SD)	Square root of the AVE^b^	Resistance	Self-threat	Age	Gender	Gender	Temporal distance	ProCap^c^	ProRec^d^
Resistance	3.49 (0.989)	0.778	(0.856)	—^e^	—	—	—	—	—	—
Self-threat	2.606 (1.324)	0.821	0.491^f^	(0.891)	—	—	—	—	—	—
Age	27.689 (8.896)	—	–0.131^g^	–0.287^f^	(—)	—	—	—	—	—
Gender	0.345 (0.476)	—	–0.073	0.058	0.107	(—)	—	—	—	—
Familiarity	1.869 (0.892)	0.841	–0.122^g^	–0.152^h^	0.162^h^	0.124^g^	(0.905)	—	—	—
Temporal distance	2.188 (0.864)	0.673	0.264^f^	0.306^f^	–0.111	0.111	–0.209^f^	(0.713)	—	—
ProCap	3.14 (1.037)	0.771	0.529^f^	0.621^f^	–0.132^g^	0.006	–0.054	0.295^f^	(0.910)	—
ProRec	2.915 (1.133)	0.798	0.383^f^	0.660^f^	–0.154^h^	–0.050	–0.108	0.232^f^	0.640^f^	(0.896)

^a^Values in table are correlations between two variables. Values in parentheses are composite reliabilities.

^b^AVE: average variance extracted.

^c^ProCap: threats to professional capabilities.

^d^ProRec: threats to professional recognition.

^e^Not applicable.

^f^Significance level: *P*<.001.

^g^Significance level: *P*<.05.

^h^Significance level: *P*<.01.

**Table 6:** Results of seemingly unrelated hierarchical regression analyses with self-threat and resistance to change as dependent variables (full model 4).

				Coefficient (SE; 95% CI)		*Z* value		*P* value
**Model 4a with dependent variable self-threat**								
	**Stage 1 (controls)**							
		Age	–0.034 (0.009; –0.052 to –0.016)		–3.650		<.001	
		Gender	–0.134 (0.134; –0.396 to 0.129)		–1.000		.39	
		Familiarity	–0.086 (0.072; –0.226 to 0.054)		–1.210		.25	
		Group (experienced and novice)	0.317 (0.218; –0.109 to 0.744)		1.460		.15	
	**Step 2 (identity threats)**							
		ProRec^a^	0.503 (0.070; 0.366 to 0.641)		7.170		<.001	
		ProCap^b^	0.372 (0.080; 0.215 to 0.529)		4.650		<.001	
	**Step 3 (Temporal distance of AI^c^, interactions)**							
		Temporal distance	0.137 (0.077; –0.014 to 0.289)		1.780		.08	
		Temporal distance x ProRec	0.291 (0.078; 0.138 to 0.443)		3.730		<.001	
		Temporal distance x ProCap	–0.203 (0.086; –0.372 to –0.034)		–2.350		.02	
	Intercept			1.071 (0.276; 0.531 to 1.611)		3.880		<.001
**Model 4b with dependent variable resistance**								
	**Stage 1 (controls)**							
		Age	–0.001 (0.009; –0.018 to 0.016)		–0.130		.90	
		Gender	–0.134 (0.127; –0.382 to 0.114)		–1.060		.29	
		Familiarity	–0.066 (0.068; –0.198 to 0.067)		–0.970		.33	
		Group (experienced and novice)	–0.061 (0.206; –0.465 to 0.343)		–0.300		.77	
	**Step 2 (identity threats)**							
		ProRec	0.055 (0.066; –0.076 to 0.185)		0.820		.41	
		ProCap	0.400 (0.076; 0.251 to 0.548)		5.270		<.001	
	**Step 3 (Temporal distance of AI, interactions)**							
		Temporal distance	0.149 (0.073; 0.005 to 0.292)		2.030		.04	
		Temporal distance x ProRec	0.087 (0.074; –0.057 to 0.232)		1.190		.24	
		Temporal distance x ProCap	–0.165 (0.082; –0.325 to –0.005)		–2.020		.04	
	Intercept			0.237 (0.261; –0.274 to 0.748)		0.910		.36

^a^ProRec: threats to professional recognition.

^b^ProCap: threats to professional capabilities.

^c^AI: artificial intelligence.

**Figure 1:**
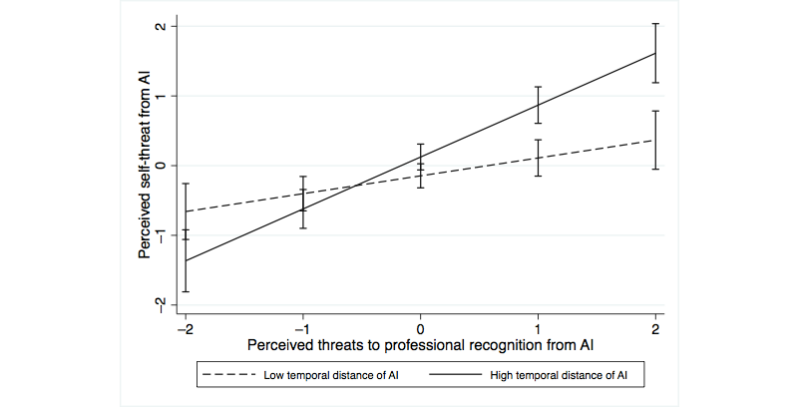
Moderating effect of temporal distance on the association of threats to professional recognition with self-threat. AI: artificial intelligence.

**Figure 2:**
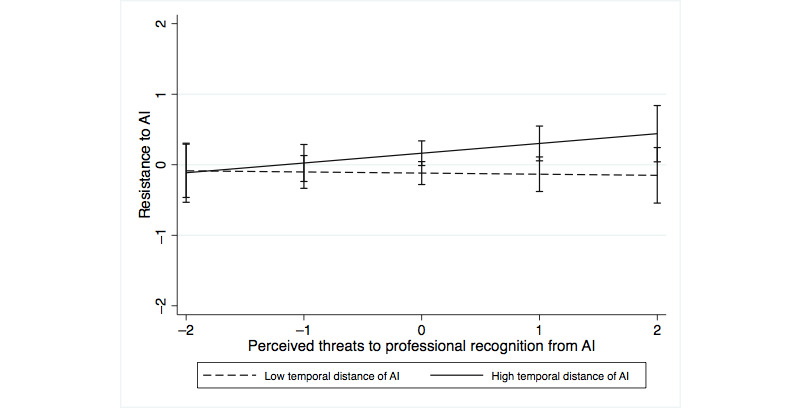
Moderating effect of temporal distance on the association of threats to professional recognition with resistance. AI: artificial intelligence.

**Figure 3:**
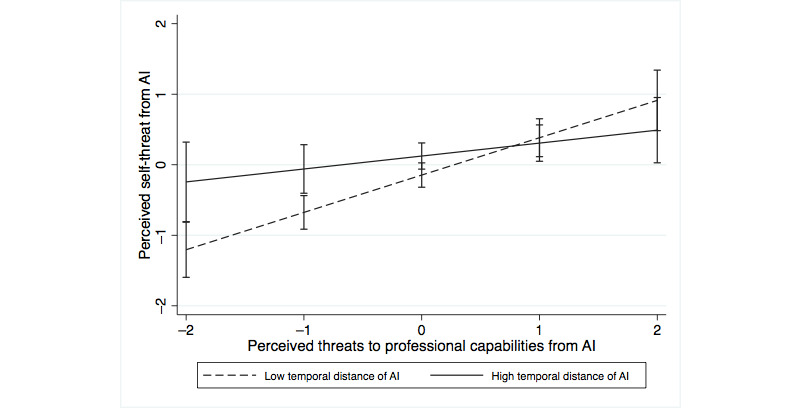
Moderating effect of temporal distance on the association of threats to professional capabilities with self-threat. AI: artificial intelligence.

**Figure 4:**
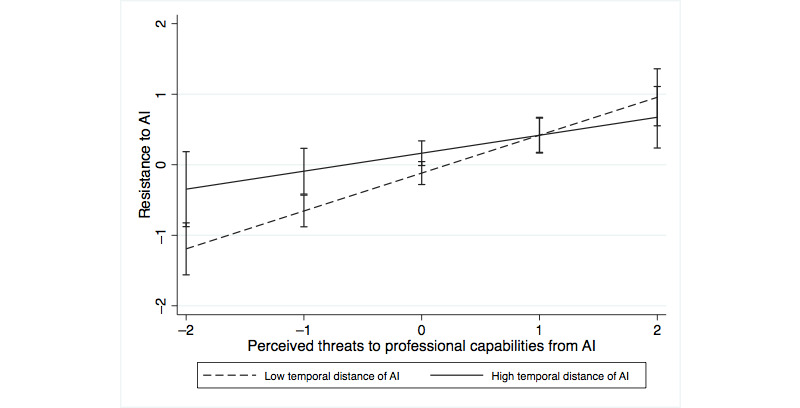
Moderating effect of temporal distance on the association of threats to professional capabilities with resistance. AI: artificial intelligence.

We also analyzed how perceiving AI systems as temporally close or distant interacted with perceived professional identity threat. The perception of AI systems as temporally distant interacted positively with threats to professional recognition (*P*<.001) and interacted negatively with threats to professional capabilities (*P=*.02) in predicting self-threat (Table 6, model 4a). In predicting resistance, the perception of AI systems as temporally distant interacted negatively with threats to professional capabilities (Table 6, model 4b; *P*=.04), whereas the interaction with threats to professional recognition was not significant (*P*=.24). Figures 1-4 show the moderating effects of temporal distance on both dimensions of identity threat. The findings suggest that experienced identity threats are closely related to how temporally distant or close the technological change from AI systems is perceived. Threats to professional capabilities refer to more concrete and context-specific elements of professional identity. Thus, these threats are more salient if physicians believe that AI systems are temporally close and relevant to clinical practice in the near future. Conversely, threats to professional recognition require physicians to consider their profession in a holistic way. Thus, these threats are more salient if physicians perceive AI systems to be relevant only in the distant future.

For the sample of novice physicians, we also collected data about perceived identity enhancements through AI. We used these data for robustness analysis to exclude the possibility that our results regarding the effects of identity threat are biased by hidden positive attitudes. The identity enhancement items mirrored the wording of the identity threat items to capture any positive expectations toward the change induced by AI systems (Multimedia Appendix 3). The analysis shows that identity enhancement reduced resistance to AI (*P*=.02) and was not related to self-threat (*P*=.52). After including identity enhancement as a control variable, the above-described effects of perceived identity threat remained qualitatively unchanged. This indicates that perceived professional identity threats have a significant effect on resistance to AI over and beyond any effects of perceived identity enhancement through AI.

Finally, we conducted a content analysis of the qualitative statements from novice physicians to validate that our measured dimensions capture the experienced changes through AI systems. The data set consisted of a total of 414 distinct statements by 176 participants. The content of all statements was classified as positive or negative statements about AI systems by 2 independent coders (EJ and one student assistant; Table 7).

**Table 7:** Content analysis of the qualitative statements.

Dimensions of identity threat and categories identified in the data		Example statements from participants (survey ID)
**Threats to professional recognition**		
	AI^a^ replaces important knowledge tasks leading to a loss in professional status	“Physicians appear to be less competent as patients believe that a small machine can also find solutions and solve their problem.” (#98) “Physicians will be replaced by computers and will only fulfill an assistant job.” (#894)
	Decreasing importance of professional knowledge and expertise	“Physicians are tempted to study less as they know that the system will have all data accessible.” (#492)
**Threats to professional capabilities**		
	Loss of professional autonomy in decision-making	“Physicians might stop to think for themselves and do not reflect on the results presented by the system which is a severe source of mistakes” (#98) “Less legal protection for the physician if he/she acts against the AI’s recommendation due to experience” (#954)
	Pressuring the role of care provider	“A physician will be more someone who operates a machine than being a doctor taking care of his/her patient’s individual needs.” (#2426) “less patient oriented care when using technology” (#850)
	Loss of influence	“The management will then require even faster decisions which results in increased time pressure.” (#719)
**Perceived enhancements**		
	AI supports decision-making by increasing decision certainty	“AI is a relief for the physician and helps to gain security in diagnosing by providing a second opinion which either encourages the physician to reflect his diagnosis a second time or strengthens the certainty of having found the right diagnosis.” (#47) “work more independently and provides them more security in their work.” (#3159)
	AI supports decision-making by helping to stay up-to-date	“it is no longer necessary to know the smallest detail of every single disease.” (#743) “AI stays abreast of the fast changes and developments in medical science.” (#250)
	AI increases workflow efficiency	“AI saves time which can then be invested in the treatment of more patients or more personalized care.” (#94)
	AI leads to better patient care	“AI increases security in diagnosing and might lead to better results. This again can increase the patient’s trust towards the physician.” (#2277)

^a^AI: artificial intelligence.

One-third of the negative statements (34/105; 32.4%) described threats to professional recognition. The implementation of AI systems was perceived as leading to a loss of status and prestige for the occupational group of physicians and made participants fear that physicians might become redundant and reduced to a mere voice of the AI system. Moreover, participants feared that expert knowledge would become less important as AI systems incorporated more up-to-date knowledge than ever possible for a human being. The statements also contained multiple threats to professional capabilities. As such, participants feared that physicians might lose their autonomy in decision-making as they might trust the AI system more than appropriate, whereas the system would perform tasks autonomously. In addition, participants perceived that it would become more difficult to be a care provider with AI systems in place, as these systems would increase the distance between physicians and patients. The participants also feared that liability issues would arise if they disagreed with AI decisions. Conversely, 3 categories of positive statements emerged from the content analysis. AI systems were perceived as supporting decision-making through reduced uncertainty and complexity in diagnostics. Moreover, they were seen as facilitators of access to knowledge, supporting especially novice physicians, by providing access to the newest guidelines and empirical findings.

## Discussion

### Principal Findings

Our work contributes to the knowledge on the impact of AI and the future of work in health care [53-55]. It shows that professional identity threats from AI systems are indeed a serious concern for novice and experienced physicians and contribute to resistance to AI. AI systems threaten both professional recognition and professional capabilities of medical professionals. Threats to professional capabilities directly contribute to resistance to AI, whereas the effect of threats to professional recognition is mediated through self-threat. Professional experience and perceived temporal distance of AI systems influence the relationship between perceived identity threats and resistance attitudes. Medical novices experience stronger identity threats than medical professionals. In addition, if AI systems are perceived as more relevant in the near future, threats to professional capabilities are more profound. If, however, AI systems are perceived as relevant in the distant future, threats to personal recognition gain in importance.

Our findings have implications for the understanding of how the medical professional identity changes with increasingly powerful AI systems and how AI systems are integrated into medical decisions. First, experienced identity threats influence how physicians adapt their professional identity to the upcoming change. For instance, study participants who indicated that “the role of the physician will be more passive, since decisions will be automated” might be less likely to choose specialties such as radiology. This can lead to fewer physicians who actively work with AI systems and develop the technological capabilities to evaluate those systems. Furthermore, several participants declared that they planned to focus on soft skills instead of analytical decision-making skills, which would rather be performed by an AI. Thus, instead of using AI systems as a second opinion and engaging in elaborate decision-making, physicians might end up delegating important tasks to AI systems without considering them in detail.

Second, threats to the professional identity cause identity protection responses [20] that directly impact technology use. In health care, physicians are pivotal for developing the ground truth for learning algorithms and for identifying relevant explanations and predictive values. Furthermore, physicians can make better diagnosis decisions with the support of trained algorithms and use them as a second opinion [56]. However, if they feel threatened in their identity, physicians are less likely to engage in the active development and adaptation of AI systems and resist their implementation. Moreover, identity protection responses can lead to incorrect medical decisions with AI systems if physicians reject AI advice as soon as it contradicts their opinion and is perceived as threatening [51]. In particular, threats to professional capabilities play a focal role in developing negative attitudes toward AI systems and should, thus, be addressed through specific medical training in interacting with AI systems.

### Limitations and Future Research

This study has several limitations that can serve as a springboard for future research. First, by using the survey method, we were not able to capture how the identity develops through a longer period of time and whether medical students who perceived stronger threats to their future from AI would switch to a *nonthreatened* profession that requires more subjective interpersonal skills. In addition, it would be interesting to see how the professional identity is affected in clinical practice through a more intensive interaction with AI systems. Furthermore, our study provides first insights into potential differences in experienced identity threats across medical specialties. Specialties such as radiology or pathology were scarce in our sample, although those specialties often use AI in medical practice. Consequently, a follow-up study that looks at differences across specialties in more detail might provide interesting insights. In addition, our sample consisted of respondents who reported a relatively low degree of familiarity with AI systems. This reflects the current situation in medical education, in which medical novices are not trained in the use of AI systems. However, whether a sample with more familiarity would experience lower degrees of threat from AI systems needs to be further researched. Second, as noted in the literature [28,33,57] and underlined by the qualitative survey responses, there are also positive appraisals of AI systems that can enhance, rather than threaten, individuals’ identity. Given that there are both strong positive and negative perceptions of the impact of AI systems on the professional identity, future research should consider the impact of ambivalence [58] on professional identity formation and restructuring. Third, we presented an AI system with a 90% accuracy rate. However, in clinical practice, the accuracy rate is highly dependent on the context, that is, the complexity of patient cases, and can be heavily disputed by medical professionals. Furthermore, with lower perceived or actual accuracy, physicians might develop more negative attitudes toward the AI system. Finally, as our study examined only 2 dependent variables, it is important to investigate how professional identity threat from AI systems impacts other variables, such as anxiety and long-term behaviors.

## Appendix

Multimedia Appendix 1 Detailed overview of prior research on identity threats.

Multimedia Appendix 2 Overview of item development process.

Multimedia Appendix 3 Additional analysis details, including confirmatory factor analysis, common method bias analysis, regression analysis details for models 2 and 3, robustness analysis with identity enhancement, and details on identity enhancement items.

## Abbreviations

AI: artificial intelligence
