# Supplementary material for: Identity Threats as a Reason for Resistance to Artificial Intelligence: Survey Study With Medical Students and Professionals
Source: JMIR Form Res. 2022 Mar 23;6(3):e28750. doi: 10.2196/28750 (PMC8987955; doi:10.2196/28750)
Supplement: Multimedia Appendix 1 [file formative_v6i3e28750_app1.docx]

# Multimedia Appendix 1. Overview of prior research

| Table S1-1. Overview of prior research considering roles of medical professionals that are threatened by the introduction of new technologies sorted by methodological approach | | | | |  |
| --- | --- | --- | --- | --- | --- |
| Study | Threatened professional roles | Dimension of identity threat | Response to the threat | Technology |  |
| Qualitative research | | | | | |
| [1] | Professional values, status loss and social identity conflict | Threats to professional recognition | Identity change and adopting new professional identity only successful if identity threats are resolved | Emerging technologies as one aspect in the larger societal change | |
| [2] | Loss of control over work practices and threat to identity | Threats to professional capabilities | Interaction between perception of technology and agency of medical professionals shape interaction with technology | Electronic patient record | |
| [3] | Professional relationships change, deskilling and general identity threat | Threats to professional capabilities | Introduction of a new technology results in a redefinition of the professional role | New technologies | |
| [4] | Professional autonomy, focus on treatment, role of guidelines and view of patients | Threats to professional capabilities | Changes result in a reconstruction of the professional role identity in interaction with the institutional structure | Introduction of an integrated information system as part of larger change | |
| [3] | Comparing against old identity; threats to “work methods and habits” | Threats to professional capabilities | Negative emotions such as frustration, anger or worry result from experienced identity threat | Electronic health record | |
| [6] | Work and economic, to status and power versus other groups | Threats to professional recognition and professional capabilities | Resistance to new technologies evolves as a response to perceived threats and can escalate from individual to group level resistance | Clinical information system | |
| [7] | Threat to group power, control and professional autonomy | Threats to professional capabilities | Resistance to the implementation of an information system in a hospital is driven by how doctors perceived the technology and their role | Computerized control system | |
| Quantitative research | | | | | |
| [8] | Care provider and community identity deterioration | Threats to professional capabilities | Negative impact on IT Assimilation | Electronic health records | |
| [9] | Professional autonomy as “control over the conditions, processes, procedures or content” | Threats to professional capabilities | Negative impact on adoption outcomes | Clinical decision support system | |
| [10] |  |  |  | Clinical decision support system and electronic health records | |
| [11] | Perceived threat to control over resources and processes | Threats to professional capabilities | Positive impact on resistance attitudes | Healthcare information system | |

**References**

1. Kyratsis Y, Atun R, Phillips N, Tracey P, George G. Health Systems in Transition: Identity Work in the Context of Shifting Institutional Logics. Acad Manag J 2016;60(2):610–641.

2. Jensen TB, Aanestad M. Hospitality and hostility in hospitals : a case study of an EPR adoption among surgeons. Eur J Inf Syst 2007;16(2):672–680.

3. Korica M, Molloy E. Making sense of professional identities: Stories of medical professionals and new technologies. Hum Relations 2010;63(12):1879–1901.

4. Chreim S, Williams BE, Hinings CR. Interlevel influences on the reconstrution of professional role identity. Acad Manag J 2007;50(6):1515–1539.

5. Nach H. Identity Under Challenge. Manag Res Rev 2015;38(7):703–725.

6. Lapointe L, Rivard S. A Multilevel Model of Resistance to Information Technology Implementation. MIS Q 2005;29(3):461–491.

7. Doolin B. Power and resistance in the implementation of a medical management information system. Inf Syst J 2004;14(4):343–362.

8. Mishra AN, Anderson C, Angst CM, Agarwal R. Electronic Health Records Assimilation and Physician Identity Evolution: An Identity Theory Perspective. Inf Syst Res 2012;23(3):738–760.

9. Esmaeilzadeh P, Sambasivan M, Kumar N, Nezakati H. Adoption of clinical decision support systems in a developing country: Antecedents and outcomes of physician’ s threat to perceived professional autonomy. Int J Med Inform 2015;84(8):548–560.

10. Walter Z, Lopez MS. Physician acceptance of information technologies: Role of perceived threat to professional autonomy. Decis Support Syst 2008;46(1):206–215.

11. Bhattacherjee A, Hikmet N. Physicians’ Resistance toward Healthcare Information Technology: A Theoretical Model and Empirical Test. Eur J Inf Syst 2007;16(6):725–737.
