# Supplementary material for: Identity Threats as a Reason for Resistance to Artificial Intelligence: Survey Study With Medical Students and Professionals
Source: JMIR Form Res. 2022 Mar 23;6(3):e28750. doi: 10.2196/28750 (PMC8987955; doi:10.2196/28750)
Supplement: Multimedia Appendix 2 [file formative_v6i3e28750_app2.docx]

# Multimedia Appendix 2. Item development process

## Measure development procedure for identity threat

The following Table S2-1 describes the measure development procedure that was applied for developing the measures for threats to professional capabilities and threats to professional recognition. The wording of the items was developed in multiple iterations with a trained psychologist (one of the authors) and experienced researchers in information systems with project experience in healthcare. Then the wording was iterated in a think-aloud task with four medical students [1]. Assessing identity threats through survey items can be challenging if participants would rather not openly confirm perceiving threats [2] or are not consciously aware of them. Therefore, the items were reworded as perceived fears [see also 3] and reworded from the first person form (“I”) to a more distant third person wording [2]. The content validity of the items and the measured constructs was assessed using the card sorting task [4]. The two dimensions were first considered as individual- and group-related threats as threats to professional recognition are more related to the individual standing while threats to professional capabilities are related to shared group norms. Through the review process, the naming of the categories was refined into threats to professional capabilities and threats to professional recognition. While the measure of threats to professional recognition has been developed based on the qualitative papers, we could use existing measures for threats to professional capabilities. Note that prior research has used a variety of scales in this regard: a 6-item *perceived threat to professional autonomy* scale [5], a 3-item *perceived care provider identity deterioration* and a 4-item *perceived physician community identity deterioration* scale [6], and as a 4-item *perceived threat* scale [7]. Yet, the items for care provider deterioration by [6] include expertise, status and power position, and the authors note that the correlation between their developed items was low, indicating that multiple constructs were measured. Also, [5] and [7] conceptualize threat to professional autonomy and threat in general as loss of control over processes. Thus, we disaggregated these measures and recombined them to assess the three elements of threats to professional capabilities more systematically and compliant with the construct definitions.

| Table S2-1. Application of steps for development of the dimensions of professional identity threats [8] | |
| --- | --- |
| Step 1: Develop a conceptual definition of the construct  | Five distinct professional roles were identified from literature review and classified according to two dimensions of professional identity threats. These dimensions were validated with practitioner interviews. |
| Step 2: Generate items to represent the construct | Items were generated based on construct definitions and existing literature and worded after multiple iterations in terms of perceived fears based on [3] |
| Step 3: Assess the content validity of the items | Content validity was established through a pretest with the card sorting method [4] and direct feedback |
| Step 4: Formally specify the measurement model | The item measures were defined to be reflective based on [9] |
| Step 5: Collect data | Data was collected with an online survey among 182 medical students |
| Step 6: Scale purification and refinement | Based on a series of exploratory factor analysis, items were dropped and two factors were established |
| Step 7: Reexamine scale properties | Additional data from medical professionals was collected and scale was refined. Two items were removed. |
| Step 8: Assess scale validity | Scale validity was assessed following [10]. Nomological validity was examined |
| Step 9: Cross-validate the scale | Cross-validation was conducted by assessing the nomological network and the qualitative survey data |

The generated items were analyzed via explorative factor analysis (EFA) using SPSS 25 with a sample of mainly novice physicians. EFA is applied to reduce the dimensions of new developed constructs and to assess convergent and discriminant validity [11]. In a sequence of four EFAs, items I4, A2, C2 and E5 were sequentially excluded from further analysis. All item loadings were significant. The comparative fit index (CFI) was 0.94, the normed fit index (NFI) was 0.90, the goodness of fit index (GFI) was 0.87, the adjusted goodness of fit index (AGFI) was 0.83 and the Trucker-Lewis index (TLI) is 0.93. The cut-off value for all these indexes is 0.90 [see also 12]. Then, we validated the remaining items with a second sample consisting of mainly experienced physicians (n=42). Items E3, S3, S5 and S6 were excluded based on an EFA due to high cross-loadings on both identified factors. The following Table S2-2 provides an overview of the items that were dropped in the process.

| Table S2-2. Dropped Items for Threats to Professional Recognition and threats to Professional Capabilities. | | | | |
| --- | --- | --- | --- | --- |
| Construct | Item | | Based on | Item development |
|  |  | |  |  |
| Perceived threat to expertise (ProRec) | E1 | I fear that when using the system, the medical expertise will not be needed anymore for patient treatment. | Self-developed | Dropped in first EFA |
|  | E3 | I fear that when using the system specialized medical skills will not be needed anymore for patient care. | [6] | Dropped in second data collection |
|  | E5 | I fear that when using the system physicians may feel less competent. |  | Dropped in first EFA |
|  |  |  |  |  |
| Perceived threat to status position (ProRec) | S3 | I fear that physicians, who use the system, may be less recognized in the physician community. |  | Dropped in second data collection |
|  | S5 | I fear that when using the system, the position of physicians’ vs non-­physicians may deteriorate. | [5] | Dropped in second data collection |
|  | S6 | I fear that the medical community as such will lose their position. | [6] | Dropped in second data collection |
|  |  |  |  |  |
| Perceived threat to professional autonomy (ProCap) | A2 | I fear that the way how physicians do their job will change in future | [6] | Dropped in first EFA |
|  |  |  |  |  |
| Perceived threat to professional influence (ProCap) | I4 | I fear that issues of data security will arise. | Self-developed | Dropped in first EFA |
|  |  |  |  |  |
| Perceived threat to  being a care provider (ProCap) | C2 | I fear that when using the system, the physician-patient relationship will deteriorate. | [6]) | Dropped in first EFA |

An exploratory factor analysis for all items of the identity threat scales resulted in two factors. In a stepwise approach, individual items were removed to ensure good scale properties. The remaining items were loading on one of two factors with high loadings on a ”parent” factor (> .60) and low loadings (< .40) on a ”foreign” factor [13]. The scales met the guidelines of [10] for convergent validity. The final measure consists of five items for threats to professional recognition and seven items for threats to professional capabilities.

A confirmatory factor analysis (CFA) with all measurement scales (n=206) resulted in a good-model fit: χ^2^ = 360.35, CFI = 0.95, TLI = 0.95, RMSEA = 0.06 and SRMR = 0.06. All scales displayed good psychometric properties including reliability, convergent and discriminant validity. The composite reliabilities (CR) of the scales were satisfactory and mainly above 0.85, only psychological distance had a CR of 0.71. The average variance extracted (AVE) for psychological distance was 0.45. For all other factors, the AVE was above 0.50. The correlation between the newly developed constructs professional recognition and professional capabilities was 0.64 which was smaller than the lowest square root of AVE of 0.77. Self-threat and professional recognition had a correlation of 0.66, but the lowest square root of AVE of self-threat was higher indicating acceptable multicollinearity. Furthermore, we accounted for potential common method bias in the survey design and through testing for the common method factor. The results indicated that the common method is unlikely to have a strong impact on our results.

**References**

1. Willis GB, Royston P, Bercini D. The use of verbal report methods in the development and testing of survey questionnaires. Appl Cogn Psychol 1991;5(3):251–267.

2. Fisher RJ. Social Desirability Bias and the Validity of Indirect Questioning. J Consum Res 1993;20(9):303–315.

3. Bick M, Kummer T-F, Ryschka S. Determining Anxieties in Relation to Ambient Intelligence—Explorative Findings from Hospital Settings. Inf Syst Manag 2015;32(1):60–71.

4. Moore GC, Benbasat I. Development of an Instrument to Measure the Perceptions of Adopting an Information Technology Innovation. Inf Syst Res 1991;2(3):192–222.

5. Walter Z, Lopez MS. Physician acceptance of information technologies: Role of perceived threat to professional autonomy. Decis Support Syst 2008;46(1):206–215.

6. Mishra AN, Anderson C, Angst CM, Agarwal R. Electronic Health Records Assimilation and Physician Identity Evolution: An Identity Theory Perspective. Inf Syst Res 2012;23(3):738–760.

7. Bhattacherjee A, Hikmet N. Physicians’ Resistance toward Healthcare Information Technology: A Theoretical Model and Empirical Test. Eur J Inf Syst 2007;16(6):725–737.

8. MacKenzie SB, Podsakoff PM, Podsakoff NP. Construct Measurement and Validation Procedures in MIS and Behavioral Research : Integrating New and Existing Techniques. MIS Q 2011;35(2):293–334.

9. Jarvis CB, MacKenzie SB, Podsakoff PM. A Critical Review of Construct Indicators and Measurement Model Misspecification in Marketing and Consumer Research. J Consum Res 2003;30(2):199–218.

10. Fornell C, Larcker DF. Evaluating Structural Equation Models with Unobservable Variables and Measurement Error. Source J Mark Res 1981;18(1):39–50.

11. Van der Heijden H. User Acceptance of Hedonic Information Systems. MIS Q 2004;28(4):695–704.

12. Iacobucci D. Structural equations modeling: Fit Indices, sample size, and advanced topics. J Consum Psychol 2010;20(1):90–98.

13. Straub D, Boudreau M-C, Gefen D. Validation Guidelines for IS Positivist Research. Commun Assoc Inf Syst 2004;13(24):380–427.
