# Supplementary material for: Identity Threats as a Reason for Resistance to Artificial Intelligence: Survey Study With Medical Students and Professionals
Source: JMIR Form Res. 2022 Mar 23;6(3):e28750. doi: 10.2196/28750 (PMC8987955; doi:10.2196/28750)
Supplement: Multimedia Appendix 3 [file formative_v6i3e28750_app3.docx]

# Multimedia Appendix 3. Factor analysis and robustness checks

## Results confirmatory factor analysis

| **Table S3-1. Overview of item loadings for model 1 (N=206) from confirmatory factor analysis** | | | | | | | |  |
| --- | --- | --- | --- | --- | --- | --- | --- | --- |
| Indicators | AVE | $\sqrt{\mathbf{AVE}}$ | 1 | 2 | 3 | 4 | 5 | 6 |
| Resistance 1 | 0.59 | 0.77 | 0.79 |  |  |  |  |  |
| Resistance 2 |  |  | 0.84 |  |  |  |  |  |
| Resistance 3 |  |  | 0.70 |  |  |  |  |  |
| Resistance 4 |  |  | 0.74 |  |  |  |  |  |
| Self-threat 1 | 0.50 | 0.71 |  | 0.49 |  |  |  |  |
| Self-threat 2 |  |  |  | 0.76 |  |  |  |  |
| Self-threat 3 |  |  |  | 0.81 |  |  |  |  |
| Self-threat 4 |  |  |  | 0.73 |  |  |  |  |
| Threats to professional recognition 1 | 0.56 | 0.75 |  |  | 0.71 |  |  |  |
| Threats to professional recognition 2 |  |  |  |  | 0.73 |  |  |  |
| Threats to professional recognition 3 |  |  |  |  | 0.80 |  |  |  |
| Threats to professional recognition 4 |  |  |  |  | 0.79 |  |  |  |
| Threats to professional recognition 5 |  |  |  |  | 0.70 |  |  |  |
| Threats to professional capabilities 1 | 0.52 | 0.72 |  |  |  | 0.73 |  |  |
| Threats to professional capabilities 2 |  |  |  |  |  | 0.60 |  |  |
| Threats to professional capabilities 3 |  |  |  |  |  | 0.80 |  |  |
| Threats to professional capabilities 4 |  |  |  |  |  | 0.75 |  |  |
| Threats to professional capabilities 5 |  |  |  |  |  | 0.76 |  |  |
| Threats to professional capabilities 6 |  |  |  |  |  | 0.79 |  |  |
| Threats to professional capabilities 7 |  |  |  |  |  | 0.60 |  |  |
| Familiarity 1 | 0.71 | 0.84 |  |  |  |  | 0.78 |  |
| Familiarity 2 |  |  |  |  |  |  | 0.73 |  |
| Familiarity 3 |  |  |  |  |  |  | 0.93 |  |
| Familiarity 4 |  |  |  |  |  |  | 0.91 |  |
| Temporal distance 1 | 0.58 | 0.76 |  |  |  |  |  | 0.77 |
| Temporal distance 2 |  |  |  |  |  |  |  | 0.79 |
| Temporal distance 3 |  |  |  |  |  |  |  | 0.73 |
| Note. Loadings shown are for the item loadings on their respective first-order constructs, AVE = average variance extracted. | | | | | | | | |

## Common Method Bias

We used multiple procedures to account for potential common method bias (CMB) [1]. First, resistance and self-threat were measured in a different part of the survey. Second, we used statistical means to assess the potential impact of CMB in our analysis. Following [2] and [1], we have included a common method variable into our confirmatory factor analysis. We fixed the variance of the factor to 1 and constrained the path coefficients from the common variable to all items to the same value. The average substantively explained variance of the focal constructs is 0.705 while the average variance explained by the common factor is 0.015. Thus the ratio is 47:1 which is similar to the ratio of [3]. We then included a marker variable with low correlation to the other variables into our model, namely a four-item measure of effort expectancy [4]. By including a variable with low correlation with the other variables, the common method variance was 14% indicating that it is unlikely that CMB is a severe concern.

## Regression analysis Model 2 and Model 3

| **Table S3-2. Results of seemingly unrelated hierarchical regression analyses with self-threat and resistance to change as dependent variables with only threats to professional recognition.** | | | | | | |  |
| --- | --- | --- | --- | --- | --- | --- | --- |
|  | Coef. | Std.Err. | z | P>z | [95%Conf. | Interval] |  |
| **Model 2a with dependent variable self-threat** | | | | | | |  |
| *Stage 1 (Controls)* | | | | | | |  |
| Age | -0.032 | 0.010 | -3.090 | 0.002 | -0.052 | -0.012 |  |
| Gender | -0.026 | 0.147 | -0.180 | 0.858 | -0.314 | 0.261 |  |
| Familiarity | -0.092 | 0.078 | -1.180 | 0.239 | -0.244 | 0.061 |  |
| Group (experienced and novice) | 0.167 | 0.241 | 0.690 | 0.489 | -0.305 | 0.639 |  |
| *Step 2 (Identity Threats)* | | | | | | |  |
| Threats to ProRec | 0.738 | 0.061 | 12.140 | 0.000 | 0.619 | 0.857 |  |
|  |  |  |  |  |  |  |  |
| *Intercept* | -1.126 | 0.358 | -3.150 | 0.002 | -1.827 | -0.425 |  |
|  | | | | | | |  |
| **Model 2b with dependent variable resistance**  *Stage 1 (Controls)* | | | | | | |  |
| Age | -0.002 | 0.010 | -0.190 | 0.853 | -0.021 | 0.017 |  |
| Gender | -0.057 | 0.138 | -0.410 | 0.678 | -0.329 | 0.214 |  |
| Familiarity | -0.066 | 0.073 | -0.900 | 0.369 | -0.210 | 0.078 |  |
| Group (experienced and novice) | -0.170 | 0.227 | -0.750 | 0.455 | -0.616 | 0.276 |  |
| *Step 2 (Identity Threats)* | | | | | | |  |
| Threats to ProRec | 0.311 | 0.057 | 5.410 | 0.000 | 0.198 | 0.423 |  |
|  |  |  |  |  |  |  |  |
| *Intercept* | -0.678 | 0.338 | -2.010 | 0.045 | -1.340 | -0.016 |  |
| Notes. ProRec: Threats to professional recognition. ProCap: Threats to professional capabilities. | | | | | | |  |

| **Table S3-3. Results of seemingly unrelated hierarchical regression analyses with self-threat and resistance to change as dependent variables with only threats to professional recognition and threats to professional capabilities.** | | | | | | |
| --- | --- | --- | --- | --- | --- | --- |
|  | Coef. | Std.Err. | z | P>z | [95%Conf. | Interval] |
| **Model 3a with dependent variable self-threat** | | | | | | |
| *Stage 1 (Controls)* | | | | | | |
| Age | -0.033 | 0.010 | -3.390 | 0.001 | -0.051 | -0.014 |
| Gender | -0.079 | 0.137 | -0.570 | 0.567 | -0.348 | 0.191 |
| Familiarity | -0.113 | 0.073 | -1.550 | 0.122 | -0.255 | 0.030 |
| Group (experienced and novice) | 0.272 | 0.226 | 1.200 | 0.229 | -0.171 | 0.715 |
| *Step 2 (Identity Threats)* | | | | | | |
| Threats to ProRec | 0.491 | 0.073 | 6.760 | 0.000 | 0.349 | 0.634 |
| Threats to ProCap | 0.431 | 0.079 | 5.450 | 0.000 | 0.276 | 0.585 |
| Intercept | 1.084 | 0.284 | 3.810 | 0.000 | 0.527 | 1.641 |
|  | | | | | | |
| **Model 3b with dependent variable resistance**  *Stage 1 (Controls)* | | | | | | |
| Age | -0.003 | 0.009 | -0.300 | 0.762 | -0.020 | 0.015 |
| Gender | -0.114 | 0.127 | -0.900 | 0.370 | -0.363 | 0.135 |
| Familiarity | -0.089 | 0.067 | -1.320 | 0.188 | -0.220 | 0.043 |
| Group (experienced and novice) | -0.057 | 0.209 | -0.270 | 0.785 | -0.466 | 0.352 |
| *Step 2 (Identity Threats)* | | | | | | |
| Threats to ProRec | 0.045 | 0.067 | 0.670 | 0.501 | -0.086 | 0.177 |
| Threats to ProCap | 0.463 | 0.073 | 6.350 | 0.000 | 0.320 | 0.606 |
| *Intercept* | 0.291 | 0.263 | 1.110 | 0.268 | -0.224 | 0.805 |
| Notes. ProRec: Threats to professional recognition. ProCap: Threats to professional capabilities. | | | | | | |

## Enhancement as robustness analysis

We included enhancement as additional variable for N=171 participants (missing data from the novice sample excluded). Both threats remained significant for self-threat after including enhancement. Similarly, threats to professional capabilities remained significant on resistance after including enhancement. The R^2^ for self-threat was .52 and for resistance .32.

| **Table S3-4. Seemingly-unrelated regression with identity enhancement variable.** | | | | | | | | |
| --- | --- | --- | --- | --- | --- | --- | --- | --- |
|  | Coef. | Std.Err. | z | P>z | Coef. | Std.Err. | z | P>z |
| Dependent variable: Self-threat | | | | | Dependent variable: Resistance | | | |
| Age | -0.027 | 0.017 | -1.570 | 0.117 | -0.016 | 0.016 | -1.040 | 0.300 |
| Gender | -0.086 | 0.154 | -0.560 | 0.577 | -0.054 | 0.137 | -0.390 | 0.695 |
| Familiarity | -0.080 | 0.079 | -1.010 | 0.312 | -0.093 | 0.071 | -1.310 | 0.189 |
| Enhancement | -0.051 | 0.079 | -0.640 | 0.521 | -0.164 | 0.071 | -2.330 | 0.020 |
| ProRec | 0.477 | 0.091 | 5.240 | 0.000 | 0.451 | 0.081 | 5.580 | 0.000 |
| ProCap | 0.492 | 0.081 | 6.030 | 0.000 | 0.048 | 0.072 | 0.660 | 0.506 |
| Intercept | -1.831 | 0.627 | -2.920 | 0.003 | -0.415 | 0.557 | -0.740 | 0.456 |

We utilized the following items for measuring enhancement. The factor analysis resulted in one factor, therefore the items were combined into an average score.

| **Table S3-5. List of items used for robustness analysis** | | | |
| --- | --- | --- | --- |
| Construct | Dimension | Item | |
| Enhancement of professional recognition | Expertise | E2 | I hope that when using the system physicians may gain in their expert status. |
|  |  | E3 | I hope that when using the system no physician specializations can be replaced. |
|  | Status | S1 | I hope that when using the system physicians’ position in the hospital hierarchy may be strengthened. |
|  |  | S2 | I hope that when using the system physicians may improve in their professional status. |
|  |  | S4 | I hope that the status of physicians, who use the system, may improve within the physician community. |
| Enhancement of professional capabilities | Autonomy | A1 | I hope that when using the system physicians’ job autonomy may be increased. |
|  |  | A3 | I hope that physicians’ diagnostic and therapeutic decisions will less be monitored by non-physicians. |
|  | Professional influence | I1 | I hope that when using the system physicians may have more control over patient medical decisions. |
|  |  | I2 | I hope that when using the system physicians may have more control over ordering patient tests. |
|  |  | I3 | I hope that when using the system physicians may have more control over the distribution of scarce resources. |
|  | Careprovider | C1 | I hope that when using the system physicians have more influence on patient care. |
|  |  | C3 | I hope that when using the system physicians are more able to treat their patients well. |

**References**

1. Podsakoff PM, MacKenzie SB, Lee JY, Podsakoff NP. Common Method Biases in Behavioral Research: A Critical Review of the Literature and Recommended Remedies. J Appl Psychol 2003;88(5):879–903.

2. Ho SY, Bodoff D. The effects of web personalization on user attitude and behavior: An integration of the elaboration likelihood model and consumer search theory. MIS Q 2014;38(2):497–520.

3. Liang H, Saraf N, Hu Q, Xue Y. Assimilation of Enterprise Systems: The Effect of Institutional Pressures and the Mediating Role of Top Management. MIS Q 2007;31(1):59.

4. Venkatesh V, Morris MG, Davis GB, Davis FD. User Acceptance of Information Technology: Toward a Unified View. MIS Q 2003;27(3):425–478.
